# Novel Deep Learning Network for Gait Recognition Using Multimodal Inertial Sensors

**DOI:** 10.3390/s23020849

**Published:** 2023-01-11

**Authors:** Ling-Feng Shi, Zhong-Ye Liu, Ke-Jun Zhou, Yifan Shi, Xiao Jing

**Affiliations:** 1 School of Electronic Engineering, Xidian University, Xi’an 710071, China; 2 Department of Mechanical and Materials Engineering, Queen’s University, 130 Stuart Street, Kingston, ON K7L 3N6, Canada; 3 School of Aerospace Engineering, Georgia Institute of Technology, Atlanta, GA 30332, USA

**Keywords:** gait recognition, deep learning, convolutional neural network (CNN), bidirectional LSTM

## Abstract

Some recent studies use a convolutional neural network (CNN) or long short-term memory (LSTM) to extract gait features, but the methods based on the CNN and LSTM have a high loss rate of time-series and spatial information, respectively. Since gait has obvious time-series characteristics, while CNN only collects waveform characteristics, and only uses CNN for gait recognition, this leads to a certain lack of time-series characteristics. LSTM can collect time-series characteristics, but LSTM results in performance degradation when processing long sequences. However, using CNN can compress the length of feature vectors. In this paper, a sequential convolution LSTM network for gait recognition using multimodal wearable inertial sensors is proposed, which is called SConvLSTM. Based on 1D-CNN and a bidirectional LSTM network, the method can automatically extract features from the raw acceleration and gyroscope signals without a manual feature design. 1D-CNN is first used to extract the high-dimensional features of the inertial sensor signals. While retaining the time-series features of the data, the dimension of the features is expanded, and the length of the feature vectors is compressed. Then, the bidirectional LSTM network is used to extract the time-series features of the data. The proposed method uses fixed-length data frames as the input and does not require gait cycle detection, which avoids the impact of cycle detection errors on the recognition accuracy. We performed experiments on three public benchmark datasets: UCI-HAR, HuGaDB, and WISDM. The results show that SConvLSTM performs better than most of those reporting the best performance methods, at present, on the three datasets.

## 1. Introduction

Gait recognition is a sub-problem of human activity recognition (HAR), which has become a hot topic in recent years. The technology has broad application prospects in human identification [1], kinematic analysis [2], indoor positioning [3], and clinical medicine [4].

Gait recognition can be achieved based on inertial sensors [5,6], pressure sensors [7], electromyographic (EMG) sensors [8], and computer vision [9,10,11]. Gait recognition based on inertial sensors allows subjects to wear a single or multiple inertial measurement units (IMUs), including triaxial accelerometers and triaxial gyroscopes, to capture acceleration and angular velocity information during the movement of subjects. Inertial signals are then analyzed to identify the gait of subjects. Compared with those methods based on pressure sensors, EMG sensors, and computer vision, IMU-based (Inertial Measurement Unit) methods have the advantages of high efficiency, portability, and low cost.

The goal of gait recognition based on inertial sensors is to extract features from inertial signals to classify the subjects’ gait types. The development of gait recognition algorithms has experienced three stages: early template-matching, machine learning, and current deep learning algorithms. At present, gait recognition algorithms based on deep learning have become the mainstream. The gait recognition algorithms based on deep learning can automatically extract features through learning from a large amount of data, and usually have higher accuracy than the algorithms using manually designed features. The multimodal time-series data collected by inertial sensors contains rich temporal and spatial information. At present, in the fields of gait recognition and HAR, most studies use a CNN to extract the spatial waveform features of gait data [12]. Some studies use a recurrent neural network (RNN) [13], gated recurrent unit (GRU) [14], or LSTM [15] to extract the time-series correlation features of gait data.

Human posture recognition based on computer vision is mainly used to recognize human posture through various feature information, such as video image sequences, human body outlines, and multi-perspectives. Recognition based on computer vision can easily obtain the trajectory, contour, and other information of human movements, but there is no way to express the details of human movement, and it is easy to identify errors due to occlusions. Compared with image recognition and other recognition methods, behavior recognition based on IMU shows its advantages of low power consumption rates, portability, and low cost, and has been widely used in medical rehabilitation, human–computer interaction, and virtual reality.

In this paper, we propose a general framework for gait recognition and HAR. Firstly, the raw inertial sensor data are preprocessed, and then we build a sequential convolution LSTM network for gait recognition using multimodal wearable inertial sensors, namely, SConvLSTM. This method combines 1D-CNN and a bidirectional LSTM network. Although the LSTM network effectively solves the problem of the gradient exploding and vanishing of a long input sequence, an overlong input sequence still leads to the degradation of LSTM performance. Therefore, we first use 1D-CNN as the automatic feature extractor to encode the local, global, and time-series features of a fixed-length accelerometer and gyroscope data. While expanding the dimension of the feature vectors, we use the pooling method to compress the length of the feature vectors, and then use the bidirectional LSTM network to extract the time-series features. The experimental results show that the proposed method effectively improves the accuracy of gait recognition.

The main contributions of this paper are as follows:

(i) A general framework for gait recognition is built, which uniformly processes the raw sensor data, and can be used for any multimodal sensor gait recognition or HAR with only a small number of modifications.

(ii) A sequential convolution LSTM network for gait recognition using multimodal wearable inertial sensors, referred to as SConvLSTM, which effectively improves the accuracy of gait recognition.

(iii) We compare our model with some based on deep learning networks, including CNN, RNN, GRU, and LSTM, and some state-of-the-art studies on UCI-HAR, HuGaDB, and WISDM datasets [16,17,18,19]. The experimental results show that the proposed method outperforms the best method on three datasets.

## 2. Methodology

**Figure 1:**
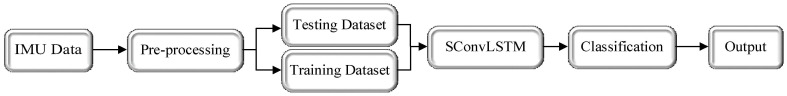
Main steps of IMU-based human activity recognition.

In this section, we elaborated the proposed SConvLSTM gait recognition network based on multimodal wearable inertial sensors. Firstly, the data preprocessing is described in Section 2.1, then the architecture of SConvLSTM is described in Section 2.2. The overall steps of our work are shown in Figure 1.

### 2.1. Pre-Processing

The key to gait recognition based on inertial sensors is to extract the features of different gait activities from acceleration and gyroscope signals. The raw accelerometer and gyroscope data contain some noise, which needs to be preprocessed. The preprocessing steps usually include denoising, normalization, and segmentation. After preprocessing, it is helpful to improve the classification and recognition accuracy of the algorithm.

#### 2.1.1. Denoising

In the process of sensor data acquisition, the shaking of a subject’s body and the deviation of the sensor’s position and direction increases the noise. The noise of the raw inertial sensor data may lead to the failure of the neural network to correctly learn features, resulting in the degradation of classification and recognition factors. Therefore, it is necessary to perform denoising. Low-pass, high-pass, and moving average filters, and other methods, can be used for denoising. This paper adopted the moving average filter. The key parameter of sliding average filtering is the length of a sliding window. The longer the window length, the smoother the filtered signal; however, increased waveform information will be lost accordingly. To ensure the integrity of waveform features while filtering, the length of the sliding window was set to 10. Here is the empirical value of the sliding filter window in recent papers: if the window is longer, the waveform will be smoother but some features will be lost.

**Figure 2:**
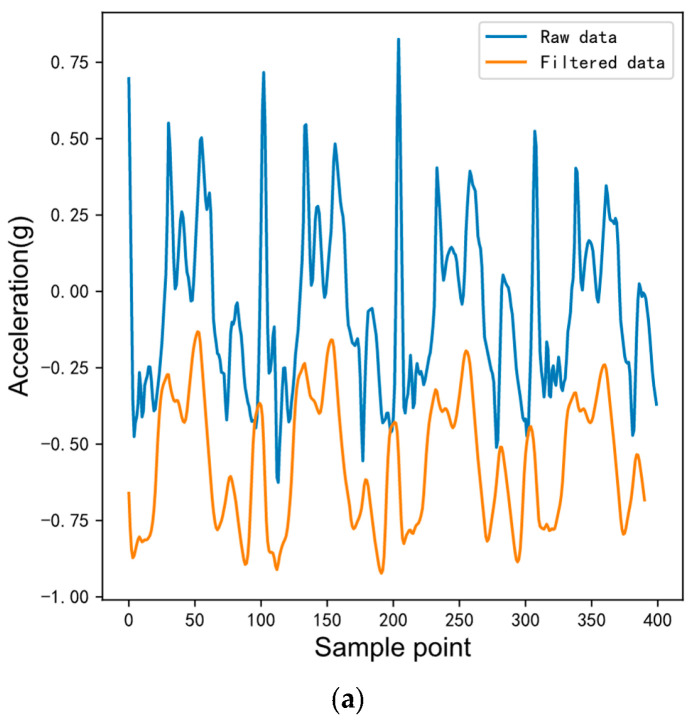

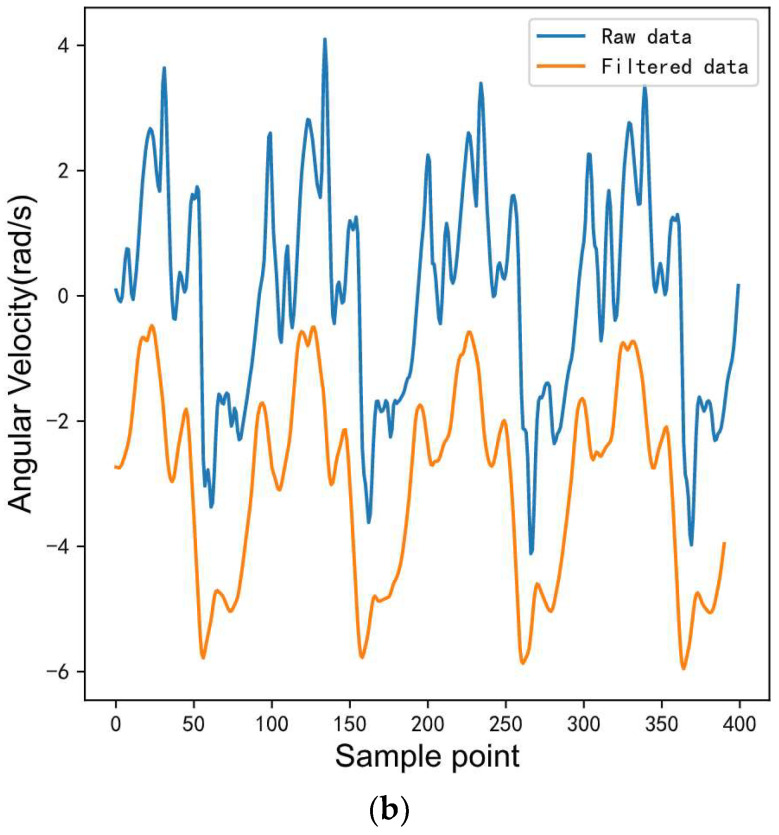
Comparison between raw and filtered IMU data. (**a**) Comparison between raw and filtered 3D acceleration data. (**b**) Comparison between raw and filtered 3D angular velocity data.

Figure 2 presents the comparison diagram of acceleration and gyroscope x-axis signals before and after filtering during walking. The x-axis is the sampling point, y-axis is the acceleration and gyroscope sampling value, respectively, and the units are specified gravities (g) and radians per second (rad/s), respectively. In order to clearly compare the difference between the raw and filtered data, the filtered data is translated in the y-axis direction. The blue lines represent the raw data and the orange lines represent the filtered data. It can be observed that the waveform becomes smoother after the moving average filtering.

#### 2.1.2. Normalization

The unit and amplitude of accelerometer and gyroscope data are quite different. It is necessary to normalization the data to produce values of all the data in the same order of magnitude. Data normalization can speed up model training. In this study, zero mean normalization was used to normalize the acceleration and gyroscope data to [−1, 1]; the formula is as follows:

(1) $$x^{*}=\frac{x-u}{\sigma }$$

where x* is normalized, *x* is the variable, *u* is the mean, and *σ* is the standard variance.

#### 2.1.3. Data Segmentation

The raw inertial sequence data are very long, and the input size of the neural network is fixed. It needs to be segmented into shorter data frames and then input into the neural network for feature extraction and classification. The segmentation methods of gait data mainly include cycle- and frame-based methods.

Cycle-based methods segment the sequence data according to the gait cycle. A gait cycle contains the features of the type of gait activities. The key step of cycle-based methods is cycle detection. Cycle detection is mainly based on peak search or cycle length estimation. Cycle-based segmentation methods need alignment and length normalization after segmentation to normalize the frame length of the gait data. Cycle-based methods present several disadvantages. Firstly, cycle detection may fail or misplace information, which directly leads to the decline in gait data quality. Secondly, the segmented data frames are of variable lengths, and interpolation is needed to normalize the lengths of the data frames. Moreover, cycle-based methods are only effective for periodic gait activities and cannot effectively segment aperiodic gait activities, such as turning, jumping, and so on.

**Figure 3:**
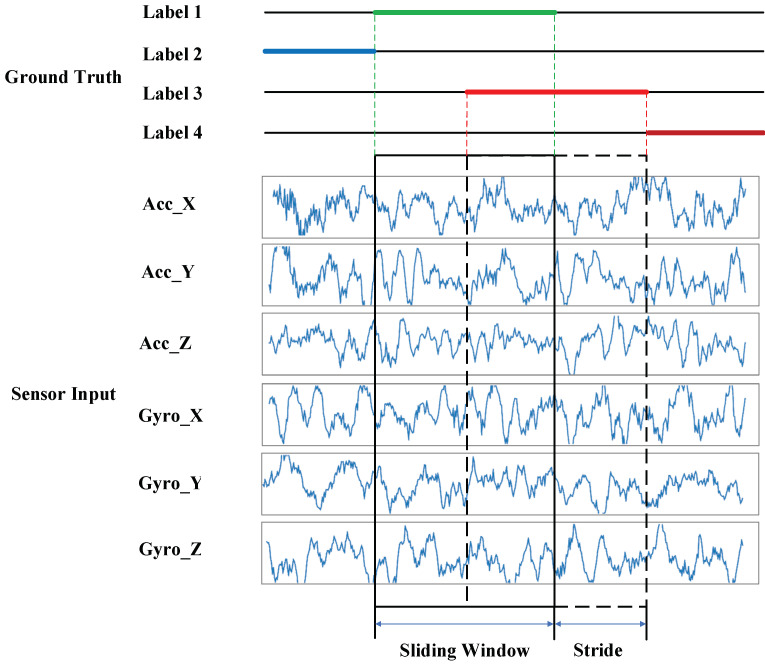
Schematic diagram of sliding window.

Frame-based methods segment the sequence data into fixed-length frames by a sliding window. There can be overlaps between the frames, which is called stride. The length of gait data frames is equal to the sampling rate multiplied by the sampling time. The sampling time is determined based on the prior knowledge of human gait activities. A single frame needs to contain at least one complete activity cycle, and the typical value is 1–5 s. In the study, a frame-based segmentation method was adopted, and the sampling time was set to 2–3 s according to different datasets. The overlap rate between data frames was set to 50%. Figure 3 shows the schematic diagram of the sliding window.

### 2.2. SConvLSTM for HAR

Multimodal inertial sensor data contains rich time-series and spatial information. In the paper, we combined 1D-CNN and bidirectional an LSTM network, and proposed a fusion architecture deep neural network model for gait recognition, referred to as SConvLSTM. The model can be used for any multimodal sensor data classification with only a small number of modifications.

#### 2.2.1. Overview

**Figure 4:**
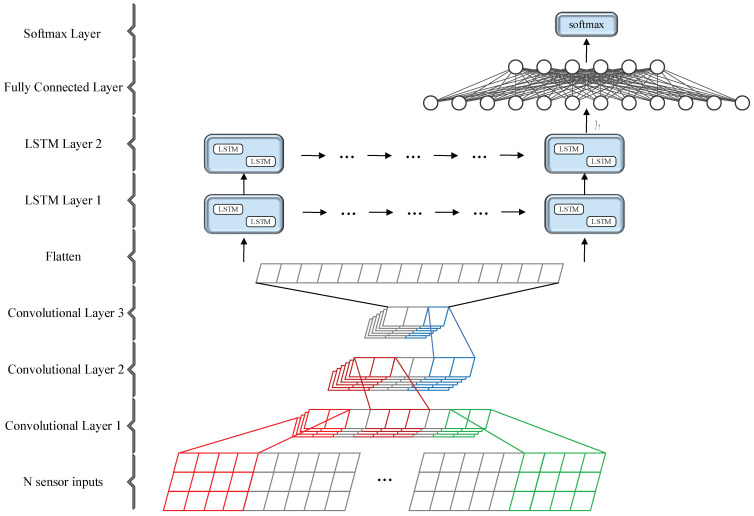
Main architecture of the proposed SConvLSTM network for human activity recognition.

The presented SConvLSTM network is mainly 1D-CNN and the bidirectional LSTM network. Figure 4 shows the overall architecture of SConvLSTM, which is mainly composed of an input part, 1D-CNN network, bidirectional LSTM network, and fully connected layer. Firstly, 1D-CNN was used to extract the high-dimensional features of the multimodal gait data. While retaining the time-series features of the data, the dimension of the features was expanded and the length of the feature vectors was compressed. Then, the convoluted high-dimensional feature vectors were input into the bidirectional LSTM to extract the time-series features.

In the input part, the fixed-length inertial data frames segmented by the sliding window were input into the network. The input data are expressed as $X\in R^{M\times N}{}^{\times T}$, where M is the total number of sensor data samples, N is the number of sensor channels, and T is the length of the gait data frames. A single input sample can be expressed as $x_{1}$,$\;x_{2}$,…,$\;x_{T-1}$,$\;x_{T}$.

1D-CNN was used to extract the high-dimensional features of the multimodal sensor data. It can expand the feature dimension and shorten the length of the feature vectors. The reason why the feature vector length is shortened is that the LSTM network is not suitable for processing overlong sequence inputs. Although LSTM effectively solves the problems of the gradient exploding and vanishing of long input sequences, overlong input sequences still lead to the degradation of LSTM, and the training and forward propagation efficiency of LSTM are significantly reduced.

The architecture of the bidirectional LSTM network is based on a two-layer bidirectional LSTM network. The output of LSTM is connected to fully connected layers. The fully connected layer maps the output of LSTM to a vector with length K, where K is the number of gait types to be classified.

#### 2.2.2. 1D-CNN

CNNs, including 1D-CNNs, 2D-CNNs, and 3D-CNNs, are widely used in the field of deep learning and artificial intelligence. 1D-CNNs are usually used for sequential signal processing, 2D-CNNs are used for image processing, and 3D-CNNs are usually used for video processing. Because the data collected by the inertial sensor are time-series signals, 1D-CNN is used as the feature extractor of the algorithm.

1D-CNN can extract the high-dimensional features of sequential data by 1D convolution. A 1D convolutional layer causes convolution by the sliding window mechanism to generate 1D activation feature maps. Following a lot of training, the model can learn the convolution kernels activated for specific features. The formula of one-dimensional convolution is shown in Equation (2):

(2) $$a_{j}^{\left(l+1\right)}(\tau )=\sigma \left(b_{j}^{l}+\sum_{f=1}^{N^{l}}K_{jf}^{l}(\tau )*a_{f}^{l}(\tau )\right)$$

where * represents the convolution operation. *a^l^_j_*(*τ*) is the feature map *j* in layer *l* and *σ* is the Sigmoid activation function. In this paper, a rectified linear unit (ReLU) was used. *N^l^* is the number of feature maps in layer *l*, *K^l^_jf_*(*τ*) is the convolution kernel of feature map *f* in layer *l*, *b^l^_j_* is the bias vector of feature map *j* in layer *l*, and *a^l^_f_*(*τ*) is the convolution of feature map *f* in layer *l*.

**Figure 5:**
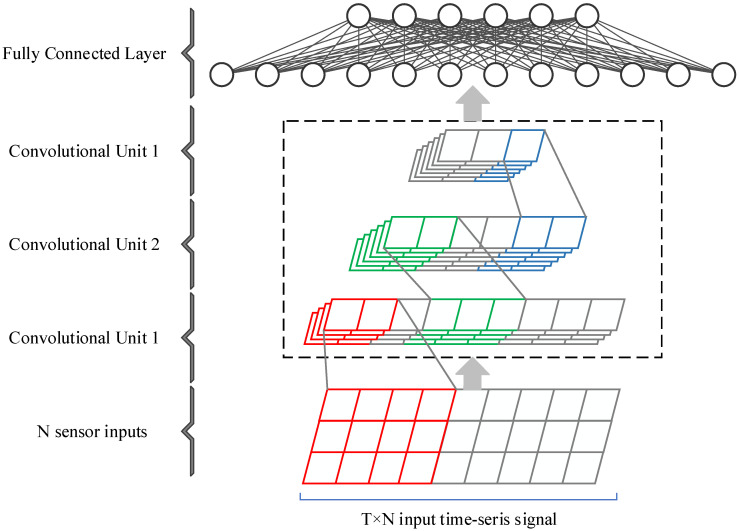
Main architecture of the 1D-CNN network.

Figure 5 shows the 1D-CNN network constructed in our study. It is mainly composed of an input part, three convolutional units, and fully connected layers. Each convolutional unit is composed of a 1D convolutional layer, a 1D batch normalization layer, a ReLU layer, and a 1D pooling layer. The length of the 1D convolution kernels is set to 5; the height is N, where N is the number of sensor channels. Due to the differences in the numbers of sensor channels of different datasets, the numbers of convolution kernels corresponding to each dataset are different. For example, in the UCI-HAR dataset, the number of convolution kernels in the first convolutional layer is 32, and the number in the second and third convolutional layers is 64. Gait recognition can be implemented only based on 1D-CNN, or 1D-CNN can be used as a feature extractor and fused with the LSTM network to improve the performance.

#### 2.2.3. LSTM

1D-CNN can effectively extract the spatial features of inertial sensor data, but it has a high loss rate of time-series information, while a recurrent neural network can extract the time-series features of sequence data. Recurrent neural networks mainly include on RNN, GRU, and LSTM.

In the RNN-based networks, the input sequence data can be expressed as $x_{1}^{l}$,$\;x_{2}^{l}$,…,$\;x_{T-1}^{l}$,$\;x_{T}^{l}$, and $h_{1}^{l}$,$\;h_{2}^{l}$, …,$\;h_{T-1}^{l}$,$\;h_{T}^{l}$ denote the activation values of the hidden layer, where *T* is the sequence length and *l* is the number of layers. On this basis, the RNN continuously uses the following Equation (3) to calculate the activation value of the hidden layer:

(3) $$h_{t}^{l}=\sigma (W_{xh}^{l}x_{t}^{l}+W_{hh}^{l}h_{t-1}^{l}+b_{h}^{l})$$

where $W_{xh}^{l}$ and $W_{hh}^{l}$ are the weight matrices of input-hidden and hidden-hidden, respectively. The activation output formula of layer *l* in the RNN is:

(4) $$x_{t}^{l+1}=W_{hx}^{l}h_{t}^{l}+b_{x}^{l}$$

where $W_{xh}^{l}$ is the hidden-activation weight matrix and $b_{x}^{l}$ is the bias vector.

**Figure 6:**
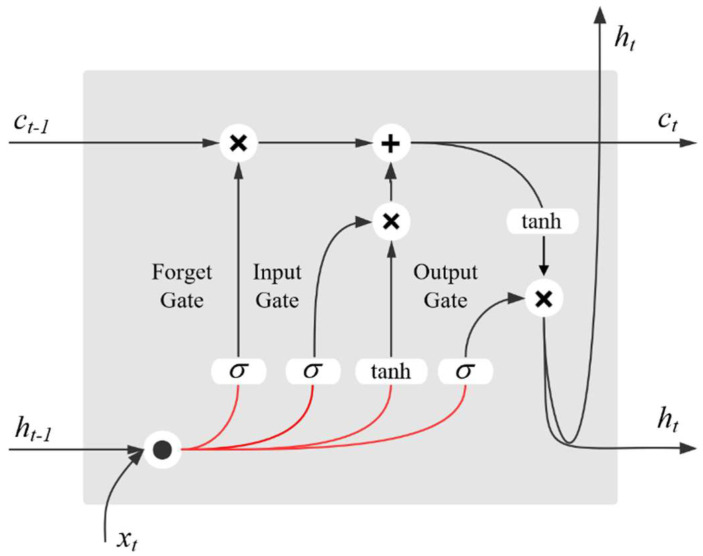
Schematic diagram of the memory cell.

On the basis that the RNN has the problems of gradient exploding and vanishing of long input sequences, LSTM effectively solves the problems through the use of a memory cell. Figure 6 is the schematic diagram of an LSTM memory cell, which is composed of input, output, and forget gates.

LSTM can learn the long- and short-term time-series features of sequence data by controlling the weight of the input, forget, and output gates. It is suitable for the prediction and classification of long-sequence data. The parameters of the memory cell are updated at each time step *t*, and the updated formulas are as follows:

(5) $$i_{t}=\sigma (W_{xi}x_{t}+W_{hi}h_{t-1}+W_{ci}c_{t-1}+b_{i})$$

(6) $$f_{t}=\sigma (W_{xf}x_{t}+W_{hf}h_{t-1}+W_{cf}c_{t-1}+b_{f})$$

(7) $$c_{t}=f_{t}c_{t-1}+i_{t}\tanh(W_{xc}x_{t}+W_{hc}h_{t-1}+b_{c})$$

(8) $$o_{t}=\sigma (W_{xo}x_{t}+W_{ho}h_{t-1}+W_{co}c_{t}+b_{o})$$

(9) $$h_{t}=o_{t}\tanh(c_{t})$$

where *x_t_* is the input at time steps *t*, *i_t_*, *f_t_*; *o_t_* and *c_t_* are the activation values of the input, forget, and output gates and self-connected memory cell at time step *t*, respectively. *h_t_* is the activation output of memory cell at time step *t*. 

**Figure 7:**
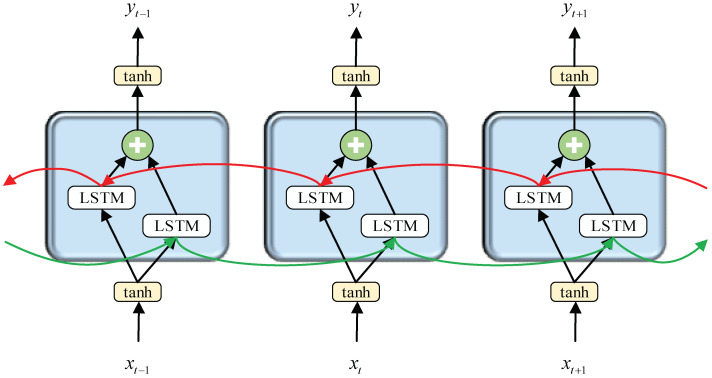
Schematic diagram of bidirectional LSTM network.

In the paper, we built a two-layer stacked bidirectional LSTM network. In the bidirectional LSTM network, the state of the current time step is related not only to the past information, but also to the future information. Figure 7 is the schematic diagram of the bidirectional LSTM network. Forward sequence $\vec{h}$ and backward sequence $\overset{\leftarrow }{h}$ exist in the hidden layer. The parameter update formulas of the bidirectional LSTM network at time t are as follows:

(10) $${\vec{h}}_{t}=\sigma (U_{\vec{h}}x_{t}+W_{\vec{h}}{\vec{h}}_{t-1}+b_{\vec{h}})$$

(11) $${\overset{\leftarrow }{h}}_{t}=\sigma (U_{\overset{\leftarrow }{h}}x_{t}+W_{\overset{\leftarrow }{h}}{\overset{\leftarrow }{h}}_{t-1}+b_{\overset{\leftarrow }{h}})$$

(12) $$y_{t}=\sigma (V_{\vec{h}}{\vec{h}}_{t}+V_{\overset{\leftarrow }{h}}{\overset{\leftarrow }{h}}_{t}+b_{y})$$

where *U*, *W*, and *V* represent the weight matrices of input-hidden, hidden-hidden, and hidden-output, respectively.

Input the sequence *x*_1_, *x*_2_, …, *x_T_*_−1_, *x_T_* into the LSTM network. The LSTM network performs *T* step iterations to extract the time-series features of the data and output *y*_1_, *y*_2_, …, *y_T_*_−1_, *y_T_*. In the classification task, *y_T_* is selected as the classification output and transferred to the following fully connected layers. Finally, the classification probability is calculated by the SoftMax function.

## 3. Experiments

To evaluate the performance of the proposed SConvLSTM network for gait recognition, the network was trained and tested on three public benchmark datasets: UCI-HAR, HuGaDB, and WISDM. The experiment was conducted on a workstation equipped with Intel Core i9 9900k CPU, NVIDIA 2070 8 G graphics card, and 16 G memory. The model was implemented using the PyTorch framework, and the code will be open source in GitHub in the future.

### 3.1. Benchmark Dataset

#### 3.1.1. UCI-HAR Dataset

The University of California, Irvine human activity recognition dataset, referred to as UCI-HAR dataset was used. The dataset was collected from the built-in MEMS IMU of a Samsung Galaxy S2 smartphone, including triaxial acceleration and triaxial gyroscope data. The dataset records the gait data of 30 subjects, including 6 gait activities: walking, going upstairs, going downstairs, sitting, standing, and lying down. The smartphone was attached to the waist, and the sampling rate was 50 Hz. The data acquisition process was recorded by video, and then the gait activities were manually labeled. The data were denoised and the gravity acceleration was filtered. To ensure that the sampling time included 1–2 gait cycles, the data were segmented into fixed-length frames by a sliding window of 2.56 s (128 points), and the stride of the sliding window was 64. There were 10,299 data samples in the dataset. We tested 10,299 samples. The publisher of the dataset divided it into training and test sets according to the ratio of 7:3, including 7352 training and 2947 test samples.

#### 3.1.2. HuGaDB Dataset

HuGaDB is one of the most comprehensive gait recognition datasets, which is divided into v1 and v2 versions. In this study, HuGaDB v2 was used for the experiments. The HuGaDB v2 dataset recorded the gait data of 18 subjects, including 10 gait activities: walking, running, going upstairs, going downstairs, sitting, and so on. The dataset was collected based on six wearable IMUs and two EMG sensors. Each IMU contained a triaxial accelerometer and a triaxial gyroscope. The six IMUs were attached to the left and right thighs, left and right calves, and left and right ankles, respectively. The EMG sensors were attached to the lateral femoral muscles. A total of 38 channel sensor signals were collected, including 36 channels of IMU sensor signals and 2 channels of EMG sensor signals. The sensor sampling rate was about 56.35 Hz. The present experiment was based on 36 channel IMU sensor signals. We segmented the data into fixed-length frames by a sliding window of 2.3 s (128 points), and the stride of the sliding window was 64. There were 17,244 samples in total after segmentation. The dataset was divided into training, verification, and test sets according to the ratio of 6:2:2.

#### 3.1.3. WISDM Dataset

The WISDM dataset was collected from the built-in triaxial accelerometer of an Android smartphone, including Nexus One, HTC Hero, and Motorola backflip. WISDM recorded the gait data of 29 subjects, including 6 gait activities: walking, jogging, sitting, standing, going upstairs, and going downstairs. The sampling rate of the accelerometer was 20 Hz. The data in this dataset were segmented by a sliding window of 10 s (200 points). A longer sliding window is conducive to improve the classification accuracy, but we believe that this will reduce the time resolution of the gait recognition algorithm. In this paper, we segmented the data by a sliding window of 3 s (60 points). After segmentation, there were 36,605 samples in total. Then, we divided it into training, verification, and tests set according to the ratio of 6:2:2.

To ensure the comparison of fixed variables, this paper used training in UCI-HAR: test = 7:3. Similarly, the ratio of the other two datasets was also set to training set:verification set:test set = 6:2:2. With the increase in the network layer, gradient disappearance and gradient explosion became more and more obvious. Compared to the other advanced papers, in order to maintain the same variables and reduce the number of convolution layers, the number of samples in the UCI-HAR and HuGaDB datasets was set to 128, and the WISDM dataset was set to 60. Table 1 shows the definitions of HuGaDB, UCI-HAR, and WIASDM.

### 3.2. Results

We comprehensively compared the proposed SConvLSTM model with some based on deep learning models, including 1D-CNN, RNN, GRU, and LSTM.

The gait recognition algorithm studied in this paper is a classification algorithm. There are multiple metrics for the performance of a classifier. The analysis based on single or partial metrics can only reflect part of the performance of the classifier. A comprehensive analysis of each metric is needed to evaluate the performance of the model. We evaluated the gait recognition model from the following metrics, including accuracy, precision, recall, F1-score, receiver operating characteristic (ROC), and area under ROC curve (AUC).

#### 3.2.1. Main Evaluation Metrics

The main evaluation metrics of the classifier include accuracy, precision, recall, and F1-score. Accuracy is defined as the ratio of correctly classified samples in all samples, and the formula is as follows:

(13) $$Accuracy=\frac{TP+TN}{TP+TN+FP+FN}$$

Table 2 shows the definitions of TP, TN, FP, and FN.

Precision is defined as the ratio of true-positive samples among all samples classified as positive. For example, for the gait of walking, precision represents the ratio of samples whose real label is walking among all samples classified as walking, and the formula is presented in Equation (14):

(14) $$Precision=\frac{TP}{TP_{}+FP_{}}$$

Recall refers to the ratio of true-positive samples among all positive samples. Recall is used to measure the ability of the classifier to correctly classify samples obtained from the positive samples. Recall is also called the true-positive rate (TPR). The formula is:

(15) $$Recall=TPR=\frac{TP}{TP+FN}$$

F1-score is the harmonic mean of precision and recall. Only when the precision and recall are high can we obtain a higher F1 score. The formula is:

(16) $$F1\text{-}score=\frac{2\times Precision\times Recall}{Precision+Recall}$$

Table 3, Table 4 and Table 5 show the classification accuracy, precision, recall, and F1-score of each deep learning model on UCI-HAR, HuGaDB, and WISDM datasets, respectively. We compared the proposed SConvLSTM model with several others based on neural network models, including 1D-CNN, SingleRNN, BiRNN, SingleGRU, BiGRU, SingleLSTM, and BiLSTM. In the experiment, the number of layers of all recurrent neural networks was 2, and the number of hidden layer units was set to 128. It can be observed that the SConvLSTM built in this paper had the highest accuracy, precision, recall, and F1-score.

#### 3.2.2. Confusion Matrix

**Figure 8:**
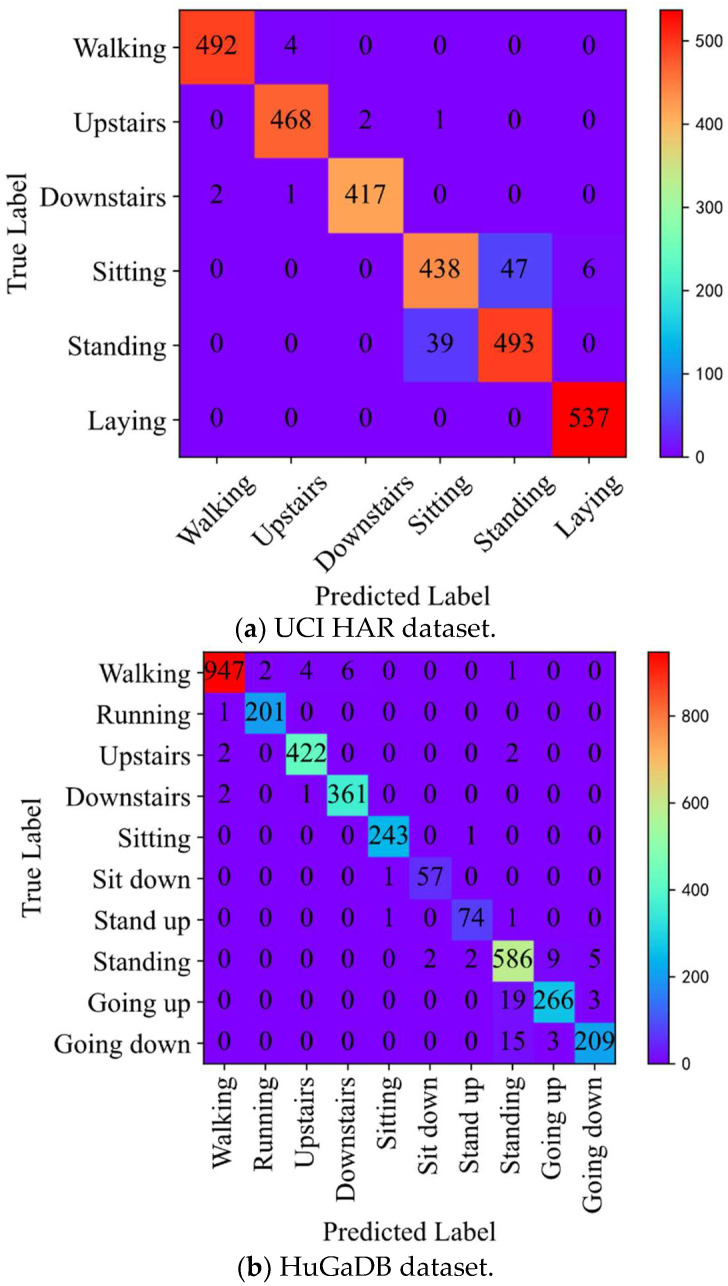

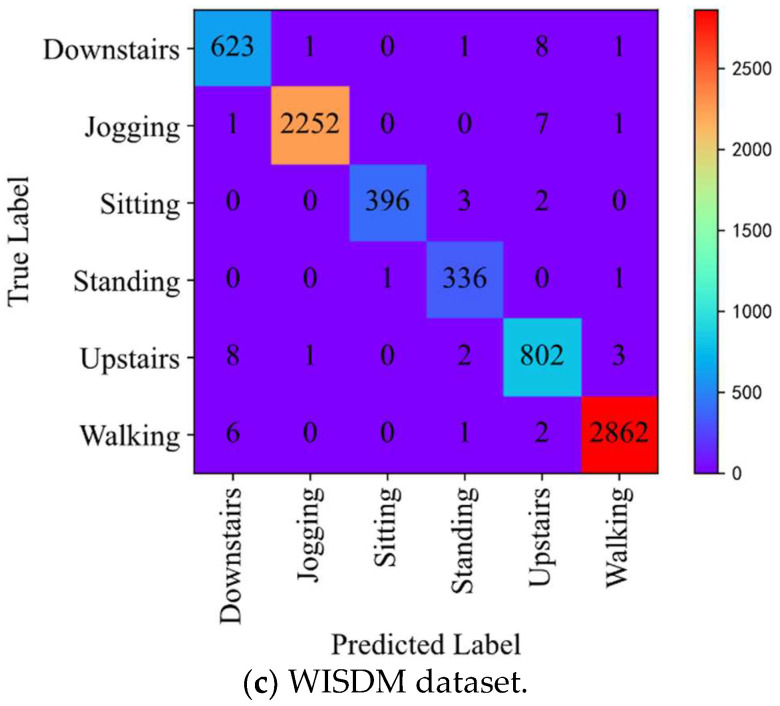
Confusion matrices for UCI HAR (**a**), HuGaDB (**b**), and WISDM (**c**) datasets.

To make a more comprehensive evaluation of the classification performance of the method proposed in the study, we analyze the confusion matrix. Figure 8 shows the confusion matrices of the proposed method on UCI-HAR, HuGaDB, and WISDM test sets. It can be observed that the presented method possesses an excellent classification performance. In the UCI-HAR dataset, there are only very few classification errors of walking, going upstairs, going downstairs, and lying down. However, there are some mistakes in the classifications of sitting and standing. We concluded that this was because, in this dataset, the smartphone collecting data were attached to the back of the subject’s waist. The sensor data for sitting and standing collected only based on a single sensor were very similar; therefore, classification confusion occurred. The final average F1-score for the UCI-HAR dataset was 96.6%. In the HuGaDB dataset, the classification of all 10 gait activities achieved high-accuracy results, with an average F1-score of 97.5%. In the WISDM dataset, there are only a few sample classification errors for the recognition of all gait activities, and the average F1-score was up to 99.3%.

#### 3.2.3. ROC Curve

**Figure 9:**
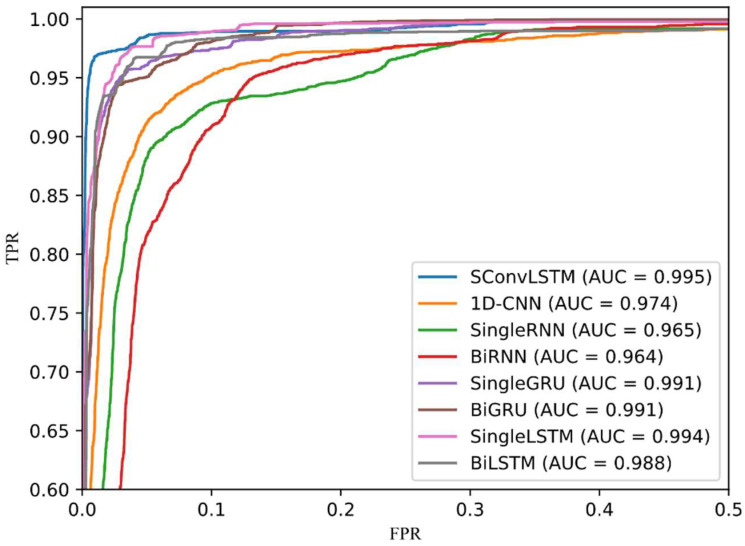
ROC curve of proposed method and some based on deep learning methods on UCI HAR dataset.

ROC curve is an evaluation curve of the statistical analysis. The x-axis is a false-positive rate (FPR) and the y-axis is a true-positive rate (TPR). The formulas for TPR and FPR are Equations (15) and (17), respectively. The ROC curve reflects the trend of the recall rate when changing the threshold of the algorithm to classify the target as a positive example. Figure 9 presents the ROC curve of all gait recognition algorithms on the UCI-HAR dataset. The curve of each color represents an algorithm, where the black, dotted line represents the random classifier. ROC curve and AUC value are calculated using confusion matrix, which can reflect the advantages and disadvantages of each algorithm in a more intuitive way. When comparing the three databases with the ROC curve and AUC value, the ROC curve overlap rate of HuGaDB and WISDM datasets is high and it is difficult to observed the comparison results. Therefore, the ROC curve comparison of UCI-HAR is presented to assist in observing the experimental results.

(17) $$FPR=\frac{FP}{FP+TN}$$

For gait recognition, we hope that the algorithm is not only accurate for gait classification, but also better for recall. As the ROC curve shows, the steeper the curve, the better the performance of the algorithm model. It can be observed from Figure 9 that our method presents the steepest curve, which indicates that its classification effect is the best. In the ROC curve, if the ROC curve of model A is on model B as a whole, model A is superior to model B. The higher the AUC value, the better the algorithm’s performance. The AUC is defined as the area under the ROC curve. The AUC value of SConvLSTM is 0.995, which is the highest value among all of the models.

#### 3.2.4. Comparison with Advanced Algorithms

We compared the classification performance of the proposed method with some state-of-the-art methods. The comparisons are based on the three public benchmark datasets: UCI-HAR, HuGaDB, and WISDM. Table 6 shows the comparative data. The publication dates, methods, accuracy, and F1-score of the relevant papers are listed. The comparative studies are the latest studies conducted since 2020, six 6 comparative studies based on the UCI-HAR dataset, two comparative studies based on the HuGaDB dataset, and five comparative studies based on the WISDM dataset.

All six studies based on the UCI-HAR dataset used deep learning methods. The accuracy of five studies was between 95–96%, and the performance was very close. Among them, the research of Zheng et al. had the best performance [23]. They proposed a model called LGSTNet. The test accuracy on the UCI-HAR dataset was 96.32% and F1-score was 95.69%. Yen et al. also achieved high accuracy based on 1D-CNN [22]. Accuracy and F1-score was 95.99% and 96.01%, respectively. When tested on the UCI-HAR dataset, the accuracy and F1-score of the model proposed in this paper were both 96.6%, which is slightly higher than the abovementioned, six recent state-of-the-art studies. Figure 9 presents the ROC curve of all gait recognition algorithms on the UCI-HAR dataset.

There are few studies based on the HuGaDB dataset. Two related studies were obtained, namely, Kumari et al.’s study in 2020 [27] and Gochoo et al.’s study in 2021 [28]. The accuracy values of their proposed methods in the HuGaDB dataset were 91.10% and 92.50%, respectively. The accuracy and F1-score of the model proposed in the paper reached 97.60%, and the accuracy was improved by 5–6%.

There are many studies based on the WISDM dataset. We selected five advanced studies in recent years for comparison. All the five studies are based on deep learning. Among them, the recognition accuracy of two studies was between 95–96%, and that of the other three studies was between 98–99%. In 2021, Tang et al. proposed a model called Lego CNN [30], which achieved the best performance on the WISDM dataset. The accuracy and F1-score were 98.82% and 97.51%, respectively. The SConvLSTM model had the highest test accuracy and F1-score of 99.3% on the WISDM dataset. Compared with Lego CNN, it has slightly improved the accuracy and F1-score in the public dataset.

## 4. Control Design and Analysis

In this paper, we proposed a gait recognition model: SConvLSTM. This model integrates 1D-CNN and the bidirectional LSTM network, which can automatically extract features without a manual feature design, which simplifies the design process of the gait recognition algorithm. The proposed method uses fixed-length data frames as inputs, and does not need gait cycle detection, which avoids the impact of cycle detection errors on recognition accuracy. We compare the proposed model with some based on deep learning models, including 1D-CNN, RNN, GRU, and LSTM. We performed experiments on three public benchmark datasets and the results show that the proposed SConvLSTM model performs well for accuracy, precision, recall, F1-score, ROC curve, and AUC value. The F1-scores of our proposed method for UCI-HAR, HuGaDB, and WISDM datasets were 96.6%, 97.6%, and 99.3%, respectively, which were significantly improved compared with the others based on deep learning models. Compared with other related studies, the SConvLSTM model shows a superior performance. Moreover, the presented method is very important, promising applications in assessing the individuals’ indoor behavior and the behavior analysis of particular patients, such as epilepsy, out-of-control behavior, and Parkinson’s disease.

## 5. Conclusions and Future Work

We presented a gait recognition model, SConvLSTM in this paper, which shows that its performance is better than other models. Moreover, the proposed method should be a very important and promising application in many fields.

The gait recognition method proposed in this paper is based on smartphone sensors, which has certain advantages in portability, and uses CNN and LSTM algorithms for fusion and complementation. This method presents classification confusion for some similar gaits, such as standing and sitting, because it is difficult to identify such similar gait activities based on a single sensor. In the future, the data obtained from multiple smart device sensors can be collected, for example, the data fusion of smartphone and smartwatch or bracelet sensors is used to improve the recognition accuracy performance.

## Figures and Tables

**Table 1:** Definitions of HuGaDB, UCI-HAR, and WIASDM.

Data Set	Population	Equipment	Location	Posture
HuGaDB	18	MPU9250, EMG	Thighs, calves, ankles	Sit, stand, go upstairs, go downstairs, walk
UCI-HAR	30	Gyroscope, accelerometer	Waist	Stand, sit, lie down, go upstairs, go downstairs
WIASDM	36	Gyroscope, accelerometer	Trouser belt	Walking, jogging, climbing stairs

**Table 2:** Definitions of TP, TN, FP, and FN.

		True Class	
		Positive	Negative
Predicted class	Positive	True Positive (**TP**)	False Positive (**FP**)
	Negative	False Negative (**FN**)	True Negative (**TN**)

**Table 3:** Classification performance of proposed method and some based on deep learning methods on UCI-HAR dataset.

Method	Accuracy	Precision	Recall	F1-Score
SingleRNN	0.850	0.853	0.850	0.849
BiRNN	0.870	0.872	0.870	0.870
SingleGRU	0.904	0.906	0.904	0.904
BiGRU	0.910	0.910	0.910	0.910
SingleLSTM	0.921	0.920	0.920	0.920
BiLSTM	0.931	0.931	0.931	0.931
1D-CNN	0.925	0.926	0.925	0.925
**SConvLSTM**	**0.966**	**0.966**	**0.966**	**0.966**

**Table 4:** Classification performance of proposed method and some based on deep learning methods on HuGaDB dataset.

Method	Accuracy	Precision	Recall	F1-Score
SingleRNN	0.831	0.822	0.831	0.824
BiRNN	0.824	0.809	0.824	0.806
SingleGRU	0.896	0.896	0.896	0.896
BiGRU	0.893	0.893	0.893	0.893
SingleLSTM	0.877	0.875	0.877	0.876
BiLSTM	0.886	0.887	0.886	0.884
1D-CNN	0.917	0.918	0.917	0.917
**SConvLSTM**	**0.976**	**0.976**	**0.976**	**0.976**

**Table 5:** Classification performance of proposed method and some based on deep learning methods on WISDM dataset.

Method	Accuracy	Precision	Recall	F1-Score
SingleRNN	0.847	0.830	0.847	0.834
BiRNN	0.795	0.752	0.795	0.757
SingleGRU	0.973	0.974	0.973	0.973
BiGRU	0.970	0.970	0.970	0.970
SingleLSTM	0.955	0.960	0.955	0.957
BiLSTM	0.974	0.975	0.974	0.974
1D-CNN	0.966	0.966	0.966	0.966
**SConvLSTM**	**0.993**	**0.993**	**0.993**	**0.993**

**Table 6:** Comparison of some state-of-the-art methods surveyed on UCI HAR, HuGaDB, and WISDM datasets.

Dataset	Year	Literatures	Method	Accuracy	F1-score
UCI HAR	2020	[20]	LSTM-CNN	95.80%	95.78%
	2020	[21]	3 Layer LSTM	93.00%	/
	2020	[22]	1D-CNN	95.99%	96.01%
	2021	[23]	LGSTNet	96.32%	95.69%
	2021	[24]	CNN-BiLSTM	96.05%	96.31%
	2022	[25]	Bi-GRU-I	95.42%	95.45%
	2022	[26]	1D-CNN	95.72%	/
	**2022**	**This work**	**SConvLSTM**	**96.60%**	**96.60%**
HuGaDB	2020	[27]	LSTM	91.10%	/
	2021	[28]	RPLB	92.50%	/
	**2022**	**This work**	**SConvLSTM**	**97.60%**	**97.60%**
WISDM	2020	[20]	LSTM-CNN	95.75%	95.85%
	2021	[29]	Capsule	95.20%	/
	2021	[24]	CNN-BiLSTM	96.37%	96.04%
	2021	[30]	Lego CNN	98.82%	97.51%
	2021	[31]	Selective Kernel Convolution	98.19%	/
	2022	[25]	Bi-GRU-I	98.25%	97.12%
	**2022**	**This work**	**SConvLSTM**	**99.31%**	**99.30%**

## Data Availability

All data are publicly available through cited, prior work.
